# The DiSCoVeR trial – first look at patient training and their expectations regarding a new, innovative treatment

**DOI:** 10.1192/j.eurpsy.2022.760

**Published:** 2022-09-01

**Authors:** L. Rubene, L. Konošonoka, M. Nahum, F. Padberg, D. Bavelier, F. Hummel, O. Bonne, E. Rancans

**Affiliations:** 1 Riga Stradins University, Department Of Psychiatry And Addiction Medicine, Riga, Latvia; 2 The Hebrew University of Jerusalem, School Of Occupational Therapy, Faculty Of Medicine, Jerusalem, Israel; 3 University Hospital, LMU Munich, Department Of Psychiatry And Psychotherapy, Munich, Germany; 4 University of Geneva, Faculty Of Psychology And Educational Sciences, Geneva, Switzerland; 5 Swiss Federal Institute of Technology, Centre For Neuroprosthetics And Brain Mind Institute, Geneva, Switzerland; 6 The Hebrew University of Jerusalem, Department Of Psychiatry, Hadassah Medical Center, Faculty Of Medicine, Jerusalem, Israel

**Keywords:** transcranial direct current stimulation, major depressive disorder, cognitive control videogame, expectations

## Abstract

**Introduction:**

The DiSCoVeR trial is a multi-site, double-blind, sham controlled, randomized controlled trial (RCT) investigating the feasibility and efficacy of an innovative, self-applied treatment approach for patients suffering from major depressive disorder (MDD). The treatment approach incorporates non-invasive brain stimulation, i.e. prefrontal transcranial direct current stimulation (tDCS), and a videogame designed to enhance emotional cognitive control. This treatment is aimed to be applied at home and monitored remotely.

**Objectives:**

In this study we are looking at the first 10 single-site patients and comparing expected in person visits (according to the study protocol) versus actual in person visits as well as looking at the patients initial view of the therapy using the therapy evaluation form (CEQ) submitted after the 5th session.

**Methods:**

Before continuing to self-administer the treatment at home patients undergo supervised training, during clinic visits, for up to 5 sessions. At the end of the 5th session, they are asked to fill out a therapy evaluation form (CEQ).

**Results:**

Patients needed on average 2.3 in person training sessions before continuing the intervention remotely. Nine patients completed CEQ. Results show that on average patients thought that this course will be 4.78 (with probability 95% CI 4.74 to 4.82) points successful at raising their level of functioning and thought that their functioning will have increased on average by 37.8% (CI 37.2% to 38.4%) by the end of the study.

**Conclusions:**

Patients needed less than half of planned in person training visits. Most patients felt like they will gain some improvement from this intervention.

**Disclosure:**

No significant relationships.

